# Evidence of age-related improvement in the foraging efficiency of Adélie penguins

**DOI:** 10.1038/s41598-019-39814-x

**Published:** 2019-03-04

**Authors:** Amélie Lescroël, Grant Ballard, Melanie Massaro, Katie Dugger, Scott Jennings, Annie Pollard, Elizabeth Porzig, Annie Schmidt, Arvind Varsani, David Grémillet, David Ainley

**Affiliations:** 1 Point Blue Conservation Science, Petaluma, CA 94954 USA; 2 Centre d’Ecologie Fonctionnelle et Evolutive, UMR 5175, CNRS - Université de Montpellier - Université Paul-Valéry Montpellier - EPHE, Montpellier, France; 3 School of Environmental Sciences, Institute for Land, Water and Society, Charles Sturt University, Albury, NSW 2640 Australia; 4 U.S. Geological Survey, Oregon Cooperative Fish and Wildlife Research Unit, Department of Fisheries and Wildlife, Oregon State University, Corvallis, Oregon USA; 5 The Biodesign Center for Fundamental and Applied Microbiomics, Center for Evolution and Medicine, School of Life sciences, Arizona State University, Tempe, Arizona USA; 6 Structural Biology Research Unit, Department of Clinical Laboratory Sciences, University of Cape Town, Observatory, Cape Town South Africa; 7 FitzPatrick Institute, DST/NRF centre of excellence at the University of Cape Town, Rondebosch, 7701 South Africa; 8 H.T. Harvey & Associates, Los Gatos, California, USA

## Abstract

Age variation in reproductive performance is well-documented but the mechanisms underlying this variation remain unclear. Foraging efficiency is likely to be a key source of demographic variation as it determines the amount of energy that can be invested in fitness-related activities. Evidence of age-related changes in the foraging efficiency of adult seabirds is scarce and inconsistent. We investigated the effects of age on the foraging efficiency of breeding Adélie penguins, a relatively short-lived seabird species, in order to gain a broader perspective on the processes driving variation in ageing rates. We found support for a positive effect of age, either linear or levelling off at old ages, on both our proxies for daily catch rate and catch per unit effort. Across all age classes, males were more performant foragers than females. We found no strong evidence for differing ageing patterns between sexes or individual quality levels, and no evidence for senescence. We infer that continuous individual improvement could be responsible for a larger amount of the variation in foraging efficiency with age at our study site, compared with selective disappearance of underperforming phenotypes. The different results reported by other studies highlight the need to conduct longitudinal studies across a range of species in different environments.

## Introduction

Age-related variation in reproductive performance and survival of iteroparous breeders is a well-documented, widespread phenomenon (e.g.^1–3^). In vertebrate populations, average performance generally increases with age in early adulthood^4,5^ as a result of population and/or individual processes. Population processes may involve the selective appearance/disappearance of individuals of different phenotypes, whereas individual processes result from improvement with age or experience (the “constraint hypothesis”) or from younger individuals reducing effort because of mortality risks (the “restraint” hypothesis). In older age classes, average reproductive performance and survival can either (1) decrease (i.e., senescence, a within-individual process predicted by mutation accumulation^6^, antagonistic pleiotropy^7^ and disposable soma^8^ theories); (2) remain constant if the disappearance of frail individuals from the population masks senescence^9^ or if high mortality levels remove individuals from the population before they begin to senesce^10^; (3) increase (reproductive performance only) as a result of terminal investment^11,12^.

The precise mechanisms underlying age variation often remain unclear and attempts to identify proximate causes, such as differences in physiology^3,13–16^ and behaviour^17–26^, have only recently been investigated in the wild. Because the ability of individuals to extract energy from their environment directly determines the amount of energy that could be invested in fitness-related activities, foraging efficiency is a key parameter to investigate. In this endeavour, seabirds have been a model of choice due to their relative longevity, size, accessibility, and the existence of long-term demographic studies. Nonetheless, evidence for age-related changes in foraging efficiency of seabirds remains relatively scarce and inconsistent across species and geographic locations, due in part to the need to couple long-term demographic data and at-sea monitoring of known individuals. Some studies found evidence for lower foraging efficiency of young, first-time breeders compared to older, experienced breeders^17,18,22–25,27^ but see^26^ on the same species^27^. A handful of studies on long-lived procellariiformes showed that old individuals had a lower foraging performance than younger individuals^17,21^. Recently, studies involving large sample sizes detected no^22^ (thick-billed murres *Uria lomvia*) or very little evidence^23^ (wandering albatrosses *Diomedea exulans*) of age-related variation in foraging behaviour, and no sign of senescence. These two studies are also the only ones, to our knowledge, to have quantified the effect of continuous age (rather than age or experience categories) on the foraging performance of breeding male and female seabirds in the wild.

It has previously been suggested that detecting senescence would be difficult in wild animals because high levels of mortality would remove individuals from the population before senescence becomes evident^10^. However, more recent comparative analyses showed that senescence is widespread in birds and mammals and that ageing rates are determined by ranking on the fast-slow life-history continuum^28^. Hence, species with a slower life history should have a lower senescence rate than species with a faster life history, for a given metabolic rate or body size. Interestingly, most studies on the age-related changes in foraging performance (defined as foraging rate or efficiency) of seabirds where senescence has been detected were conducted on species at the slow end of this fast-slow life-history continuum. Here, we investigate the effect of age on foraging efficiency of a seabird that lives a shorter life in harsher environments, the Adélie penguin (*Pygoscelis adeliae*, mean life expectancy 12–14 years, Dugger *et al*. unpubl. data, *vs*. 33 years for the wandering albatross)^29^. Indeed, Adélie penguins exhibit high fecundity and relatively low adult survival^30^ for a pelagic seabird, presumably driven by abundant seasonal food resources and high levels of predation on adults^31^. Some populations range widely at sea^32^, though some to a lesser degree^33,34^ and spend most of their lives there, yet often raise two chicks, a life-history pattern that is uncommon among pelagic seabirds^31^. Given their relatively fast life-history, we would expect to see faster aging rates in Adélie penguins compared to other seabirds.

**Figure 1:**
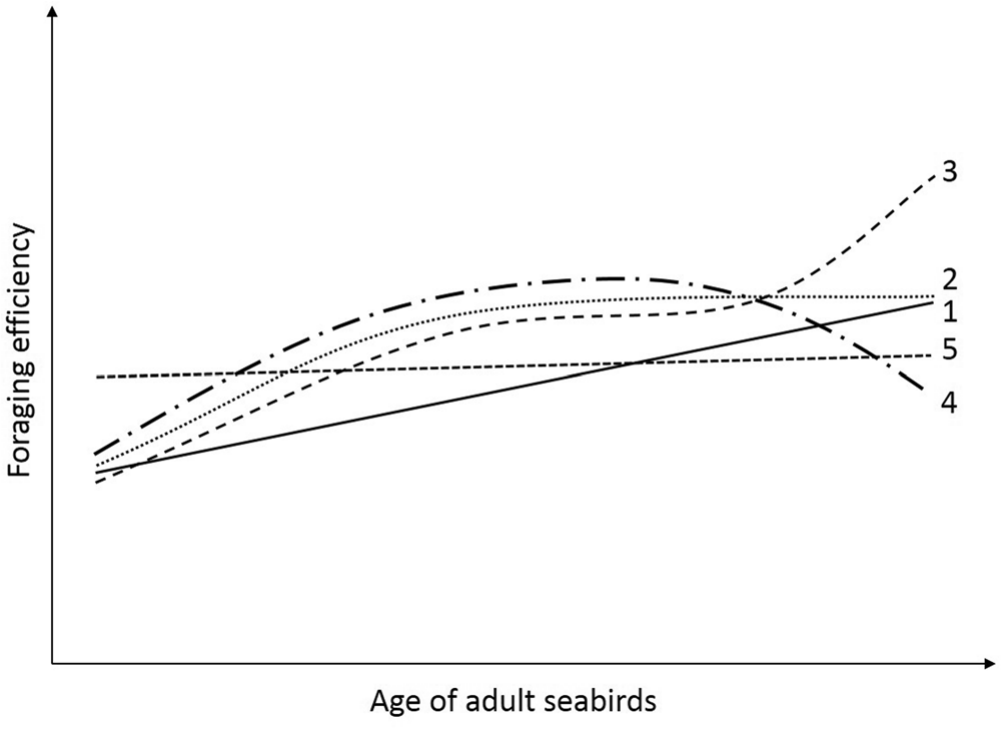
Schematic representation of the hypotheses regarding the shape of the age-specific foraging efficiency curve in sexually-mature seabirds. Foraging efficiency 1: linearly increases with age; 2: only increases in younger age classes then reaches a plateau; 3: increases in younger age classes, reaches a plateau for middle-aged individuals, then increases in older age classes; 4: increases in younger age classes, reaches an optimum for middle-aged individuals, then decreases in older age classes; 5: is constant through age classes. We investigated different intercepts and slopes (ageing rates) for each of these shapes, depending on sex, breeding quality and environmental conditions.

In the present cross-sectional study, we investigated the linear and non-linear effects of age on the foraging efficiency of breeding Adélie penguins, using proxies for daily catch rate and catch per unit effort derived from acceleration and dive data. More specifically, we tested the following hypotheses regarding the shape of the age-specific foraging efficiency curve (Fig. 1): Foraging efficiency (1) linearly increases with age (maturation or progressive selection of more efficient individuals, no senescence); (2) increases in younger age classes then reaches a plateau (pseudo-threshold response with no senescence); (3) increases in younger age classes, reaches a plateau for middle-aged individuals, then increases in older age classes (terminal investment); (4) increases in younger age classes, reaches an optimum for middle-aged individuals, then decreases in older age classes (senescence); or (5) is constant through age classes. As ageing patterns could differ between birds of different sex or different intrinsic quality, we also included interactions between age and sex, and between age and an age-independent index of breeding quality^35^. Once we had determined the most competitive models for foraging efficiency based on intrinsic characteristics of the birds (age, sex, quality), we modelled the additional effect of extrinsic variables (day in the season, year) to account for environmental variation.

## Methods

### Species and Study Site

**Figure 2:**
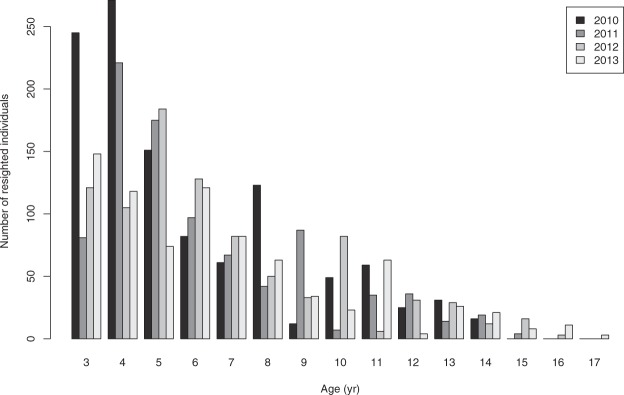
Age frequency distribution of the banded Adélie penguin population at Cape Crozier, Ross Island in 2010–2013.

Adélie penguins are pelagic seabirds, which spend only 10% of their life on land, where they form breeding colonies distributed around the Antarctic coast and high latitude offshore islands^31^. The study colony, Cape Crozier (77°27′S, 169°12′E), Ross Island, is one of the largest for this species (~275 000 pairs)^36^, and therefore exhibits significant intraspecific competition for food (Ainley *et al*. 2004; Ballance *et al*. 2009; Ford *et al*. 2015). Predation by leopard seals (*Hyrurga leptonyx*) makes foraging activity hazardous for Adélie penguins year-round^37,38^. Accordingly, Adélie penguins exhibit relatively low adult survival rates for pelagic seabirds (68–88% at Cape Crozier)^30^, and delayed maturity, with age at first breeding ranging 3–7 years for females and 4–8 years for males^31^. An improvement of reproductive success with age among young breeders (from 3- to 7-yr-old) was reported from earlier banding data^31^. Banding studies have shown that some individuals reach 20+ years of age, but few individuals reach the mid-teen years^31^ (see also Fig. 2).

Breeders arrive at Cape Crozier in late October/early November, lay (usually two) eggs in mid-November, and feed their chicks between mid-December and early February. During the guard stage, one parent remains with the chick(s) while the other forages at sea. Nest reliefs at Crozier occur every 1–2 days during early chick-rearing and chicks are fed relatively small meals by the attending parent. Afterwards, chick demands are too great for adequate provisioning by one parent, so chicks are left on their own (“crèche” stage) while both parents forage simultaneously. We investigated the effect of age on foraging efficiency during the guard stage of four austral summers (2010–2011 to 2013–2014), from mid-December to early January. Hereafter, we refer to austral summers as seasons, using their initial year (e.g., 2010 refers to the 2010–2011 breeding season).

All penguin survey, capture and handling methods used for data collection were performed following all relevant guidelines and regulations under the approval and oversight of Oregon State University’s Institutional Animal Care and Use Committee. Additionally, all work was approved and conducted under Antarctic Conservation Act permits issued by the US National Science Foundation and the U.S. Antarctic Program, and administered by H.T. Harvey & Associates.

### Population and Individual Monitoring

From 1996 to 2011, 1000 chicks, and 500 chicks from 2012 onwards, have been banded each year on the left flipper with a numbered stainless steel band (except in 2001 when only 110 chicks were banded due to very low reproduction that year; see^39^ for details on band design and effect or lack thereof on foraging behaviour). From 1997 onwards the entire colony was searched annually, on 2- to 7-d intervals (depending on the area) for banded individuals throughout the breeding season, 15 December to 25 January, and 15 November to 25 January beginning in 2002. Bands were read with binoculars from a distance (<10 m). When a banded bird was found breeding (i.e. having a nest with at least one egg or chick), its nest contents was subsequently monitored with binoculars every 2–7 d until chicks crèched (about the first week of January).

### Instrumentation

Every season, we randomly selected birds of all available ages among this pool of breeders, paying attention to keep a balanced sex-ratio as much as possible and to time the device deployments appropriately so as to always have birds of different ages and different sexes equipped at the same time. This resulted in attaching time-depth recorders (Mk9, Wildlife Computers, Redmond, Washington, USA; 68 × 17 × 17 mm, mass 30 g; hereafter called TDRs) or acceleration-time-depth recorders (G6A, CEFAS Technology Limited, Lowestoft, UK; 40 × 28 × 15 mm, mass 18 g; hereafter called accelerometers) to a total of 45 birds in 2010, 48 birds in 2011, 46 birds in 2012, and 52 in 2013. Sex was assessed in the field based on extensive observations over the lifetimes of the individuals, comparing relative size of especially head and bill, courtship behaviour (male ecstatic displays and copulation), and also timing of attendance in the colony (which is highly synchronous by sex, particularly during incubation and brood stages)^31^. Post-hoc molecular sexing was also conducted as described below.

From Dec 19–21 to Jan 2–6 (depending on years), birds were caught by hand on their nests and devices were attached to the lower back using black Tesa® tape (Hamburg, Germany)^40^, following techniques previously shown to have no detectable impacts on foraging trip duration and reproductive success^41^. Handling lasted <10 min and devices were recovered after one foraging trip at sea, lasting between 0.5–9.2 d. Due to device failure or nest failure before the completion of the foraging trip, we recovered a total of 171 files with usable data (47 TDR data files and 124 accelerometer data files from 85 females and 86 males, age 4–17 yrs, Table 1). TDRs were programmed to record pressure (with a resolution of 0.5 m), temperature and light at 1 Hz, while accelerometers recorded pressure (with a resolution of 0.08 m) and temperature at 1 Hz and 3-axis acceleration at 20 Hz. Of the 124 accelerometer data files, 10 of them failed to record acceleration but contained complete pressure and temperature data.

**Table 1:** Distribution of the 171 collected data files according to the birds’ age and sex.

Sex	Age (yr)						
	4–5	6–7	8–9	10–11	12–13	14–15	16–17
Female	24	14	17	13	12	4	1
Male	11	16	16	19	14	8	2

Diving data were processed using the program divesum (v.7.5.5; G. Ballard, unpublished software, see Appendix A in Lescroël *et al*.^42^ for more details on dive-data processing and analysis). This program corrected the record baseline and computed several individual dive parameters such as maximum dive depth, post-dive interval and number of undulations (number of changes in underwater swimming direction from ascent to descent >1 m, involving at least 3 data points, thus 3 sec).

### Measuring Foraging Efficiency

We evaluated foraging efficiency in two ways: (1) catch per unit time (trip duration) or daily catch rate, and (2) catch per unit effort (overall dynamic body acceleration).

As a proxy for prey catch, we used the total number of undulations per foraging trip, which has been shown to be linearly related to the number of prey captures^43,44^ for both fish- and krill-eating penguins feeding in the water column (as opposed to benthic feeders), such as Adélie penguins. We acknowledge that this proxy did not allow us to quantify the exact number of prey captures, as a small amount of undulations could occur without prey capture and vice-versa^45,46^. It is nonetheless a useful index to compare foraging success between individuals, in terms of relative number of prey capture events, as long as individuals are targeting the same prey in terms of size and energetic content (see Discussion).

Trip duration (from all data files) was measured from entrance in the water to exit, based on the temperature and dive profiles. Trip duration positively correlates with total energy expenditure during foraging by chick-rearing Adélie penguins (r² = 0.88)^47^.

Vectorial dynamic body acceleration (VeDBA, in units of gravitational force g, from accelerometer data only) is a measure of body motion derived from measurements of acceleration made in all 3 spatial axes^48^. This measure of the mechanical work performed by individuals can be used as a proxy for energy expenditure under certain conditions^49^. Raw acceleration values are composed of a dynamic component and a static component. The static acceleration was estimated by smoothing the data in each channel with a running mean over a period of 2 seconds, following methods by^50^. The dynamic acceleration was derived as follows: $VeDBA=\,\sqrt{{({A}_{x}-{S}_{x})}^{2}+{({A}_{y}-{S}_{y})}^{2}+{({A}_{z}-{S}_{z})}^{2}}$, where acceleration (A) is the sum of static acceleration (S) resulting from body angle with respect to gravity and dynamic acceleration (D) resulting from body movements along all three spatial axis (x: antero-posterior, y: lateral, z: vertical), i.e. A = S + D. The total mechanical power used within the trip is alluded to by measurements of VeDBA summed over the entire trip duration (cf.^48,51^).

Based on these measures, we evaluated foraging efficiency as: (1) the total number of undulations per trip divided by trip duration to get undulations per day (Uperday), as an index representing catch per unit time; and (2) the total number of undulations per trip divided by total VeDBA to get undulations per g (Uperg), as the catch per unit effort.

### Intrinsic and Extrinsic Covariates

We considered age, sex, and an age-independent index of breeding quality as intrinsic covariates that could influence foraging efficiency or the relationship between foraging efficiency and age. Age was determined by linking an individual’s cohort-specific band number to our banding database. Sex of the equipped birds was determined from blood or feather samples collected upon detachment of the devices^52^. Using breeding propensity and success data from our monitoring program, we calculated a Breeding Quality Index (BQI) for each known age individual, based on methods we developed previously^35,42^ for birds of known breeding history but unknown age. We first calculated a probability of breeding success for each year and individual using four independent variables (age, previous breeding experience, colony of origin and breeding year). The BQI of each individual was then calculated as the mean per individual of the difference between the actual breeding success and the predicted breeding success for every year during which a given individual had been resighted when at least 3 yr-old, up to the year when we equipped it with a TDR or accelerometer. More details about the BQI calculation can be found in^53^. Negative BQI values indicate lower-than-average long-term breeding performance, while positive values indicate above average long-term breeding performance. BQI for the equipped birds ranged −0.32 to 0.93.

We also considered variation of environmental conditions within and between seasons. At Cape Crozier, foraging range increases with time, requiring longer commutes between the colony and high quality prey patches^47–56^. Our previous work supports the hypothesis that conditions become more challenging as the breeding season progresses due to prey depletion by foraging Adélie penguins and cetaceans near to the colony^54,55,57^. At the same time, because Adélie Penguins are highly synchronous breeders^31^, offspring needs increase consistently among all parents. Thus, as a proxy for the within-season variation in environmental conditions, we used “study day” (a continuous variable with 0 = 20 December) and evaluated its effects on foraging efficiency. We used “year” as a factor to account for any between-season variation, with 2010 as the reference level.

### Statistical Analyses

To test our five main hypotheses (Fig. 1), we evaluated models including each foraging efficiency index (Uperday or Uperg) as the dependent variable and either a linear, pseudo-threshold (−1/Age), quadratic (Age + Age²) or cubic (Age + Age² + Age^3^) effect of age as independent variables, as well as a null model (intercept only). As ageing patterns could differ between birds of different sex or different intrinsic quality, we also included interactions between age and sex, and between age and BQI. Once we had determined the most competitive models for foraging efficiency based on intrinsic characteristics of the birds (age, sex, quality), we added extrinsic variables (study day, year) to the top intrinsic model(s) including potential interactions with age. A null model was also included in this second model set. Residuals were examined to verify normality, homogeneity of variances, and independence.

To evaluate these models and determine the strength of evidence supporting specific effects, we used an information theoretic approach^58^. Models were ranked using the small-sample-size corrected version of Akaike Information Criterion (AICc), with the best model having the lowest AICc value. We calculated ΔAICc as the difference in AICc between each candidate model and the model with the lowest AICc value, and considered all models within 2 ΔAICc as competitive models (Burnham & Anderson, 2002). We determined the strength of evidence supporting specific effects by examining the unstandardized effect sizes (slope coefficients and differences in means) and the associated 85% confidence intervals (CI), as 85% CI are more compatible with an information theoretic approach using AIC or AIC_*c*_ than 95% CI^59^. If the 85% CI for a parameter in a competitive model (ΔAIC_*c*_ < 2.0) included zero, it was considered uninformative^59^. Only models performing better than the null model and within 2 ΔAICc of the best model are reported in Tables 2 and 3.

**Table 2:** Modelling the foraging efficiency (in terms of number of undulations per day) according to intrinsic and extrinsic variables for breeding Adélie penguins at Cape Crozier, Ross Island, 2010–2013.

No.	Model	ΔAICc	K	Deviance	R²
	*Undulations per day ~ Intrinsic variables (n* = *171)*				
1	**InvAge + Sex**	**0**	**4**	**2511.663**	**0.09**
2	**Age + Sex**	**0.293**	**4**	**2511.957**	**0.08**
3	InvAge * Sex	**1.486**	**5**	**2511.027**	**0.09**
4	**InvAge + Sex + BQI**	**1.559**	**5**	**2511.099**	**0.09**
5	**Age + Sex + BQI**	**1.633**	**5**	**2511.173**	**0.09**
6	Age * Sex	**1.748**	**5**	**2511.288**	**0.09**
	Age² + Sex	2.122	5	2511.662	0.09
	InvAge * BQI + Sex	2.621	6	2510.012	0.09
	Age * Sex + BQI	3	6	2510.391	0.09
	InvAge * Sex + BQI	3.001	6	2510.391	0.09
	Age * BQI + Sex	3.044	6	2510.435	0.09
	Age² + Sex + BQI	3.645	6	2511.036	0.09
	Sex	4.174	3	2517.935	0.05
	Age^3^ + Sex	4.253	6	2511.643	0.09
	InvAge	4.42	3	2518.181	0.05
	Sex + BQI	4.925	4	2516.588	0.06
	Age	5.039	3	2518.8	0.05
	Age^3^ * Sex	5.29	9	2506.071	0.12
	Age² * Sex	5.459	7	2510.674	0.09
	Age^3^ + Sex + BQI	5.795	7	2511.01	0.09
	InvAge + BQI	6.49	4	2518.154	0.05
	Age^3^ * Sex + BQI	6.536	10	2505.059	0.12
	Age²	6.684	4	2518.348	0.05
	Age² * Sex + BQI	6.967	8	2509.979	0.09
	Age + BQI	7.039	4	2518.703	0.05
	InvAge * BQI	7.045	5	2516.585	0.06
	Age² * BQI + Sex	7.203	8	2510.214	0.09
	Age * BQI	8.033	5	2517.573	0.05
	Age^3^	8.705	5	2518.245	0.05
	Age² + BQI	8.772	5	2518.312	0.05
	Age^3^ * BQI + Sex	10.629	10	2509.151	0.10
	Age^3^ + BQI	10.813	6	2518.203	0.05
	Null	11.072	2	2526.906	0.00
	Age² * BQI	11.805	7	2517.019	0.06
	BQI	12.842	3	2526.603	0.00
	Age^3^ * BQI	15.299	9	2516.08	0.06
	*Undulations per day ~ Best intrinsic model* +*/*× *Extrinsic variables (n* = *171)*				
7	**InvAge + Sex + StudyDay**	**0**	**5**	**2508.492**	**0.10**
8	**Age + Sex + StudyDay**	**0.148**	**5**	**2508.639**	**0.10**
9	**Age + Sex + StudyDay + Year**	**0.235**	**8**	**2502.197**	**0.14**
10	**InvAge + Sex + StudyDay + Year**	**0.312**	**8**	**2502.274**	**0.13**
11	**Age + Sex + Year**	**0.336**	**7**	**2504.502**	**0.12**
12	**InvAge + Sex + Year**	**0.341**	**7**	**2504.506**	**0.12**
13	**InvAge + Sex**	**1.049**	**4**	**2511.663**	**0.09**
14	**Age + Sex**	**1.342**	**4**	**2511.957**	**0.08**
	InvAge*StudyDay + Sex	2,103	6	2508,445	0,10
	Age*StudyDay + Sex	2,284	6	2508,626	0,10
	InvAge*StudyDay + Sex + Year	2,392	9	2502,124	0,14
	Age*StudyDay + Sex + Year	2,4	9	2502,132	0,14
	Age*Year + Sex	2,678	10	2500,151	0,15
	Age*Year + Sex + StudyDay	2,951	11	2498,138	0,16
	InvAge*Year + Sex	4,145	10	2501,619	0,14
	InvAge*Year + Sex + StudyDay	4,331	11	2499,517	0,15
	StudyDay	6,183	3	2518,896	0,05
	StudyDay + Year	7,799	6	2514,141	0,07
	Year	11,998	5	2520,489	0,04
	Null	12,121	2	2526,906	0,00

Only models performing better than the Intercept-only model and within 2 ΔAICc of the best model, in bold, are numbered. InvAge = −1/Age, BQI = Breeding Quality Index. *Lowest AICc* = *2519.906 for Model 1 and 2518.857 for Model 7*.

**Table 3:** Modelling the foraging efficiency (in terms of number of undulations per unit of gravitational force g) according to intrinsic and extrinsic variables for breeding Adélie penguins at Cape Crozier, Ross Island, 2010–2013.

No.	Model	ΔAICc	K	Deviance	R²
	*Undulations per g ~ Intrinsic variables (n* = *114)*				
15	**Age**	**0**	**3**	**2060.784**	**0.03**
16	**InvAge**	**0.257**	**3**	**2061.041**	**0.03**
17	**Age + Sex**	**0.495**	**4**	**2059.13**	**0.04**
18	**InvAge + Sex**	**0.829**	**4**	**2059.464**	**0.04**
19	**Age + Sex + BQI**	**0.881**	**5**	**2057.328**	**0.06**
20	**Age + BQI**	**1.057**	**4**	**2059.692**	**0.04**
21	InvAge * Sex	**1.108**	**5**	**2057.555**	**0.06**
22	**Sex**	**1.116**	**3**	**2061.9**	**0.02**
23	Age * Sex	**1.243**	**5**	**2057.69**	**0.06**
	Intercept only	1.326	2	2064.22	0.00
	InvAge * BQI	1.359	5	2057.805	0.05
	InvAge + Sex + BQI	1.361	5	2057.807	0.05
	Age * Sex + BQI	1.366	6	2055.582	0.07
	Sex + BQI	1.398	4	2060.033	0.04
	InvAge + BQI	1.428	4	2060.063	0.04
	InvAge * Sex + BQI	1.453	6	2055.67	0.07
	Age * BQI	1.61	5	2058.056	0.05
	InvAge * BQI + Sex	1.791	6	2056.008	0.07
	Age * BQI + Sex	1.917	6	2056.134	0.07
	Age²	2.143	4	2060.778	0.03
	BQI	2.397	3	2063.181	0.01
	Age² + Sex	2.655	5	2059.101	0.04
	Age² + Sex + BQI	2.988	6	2057.205	0.06
	Age² + BQI	3.204	5	2059.651	0.04
	Age^3^	3.535	5	2059.982	0.04
	Age^3^ + Sex	4.284	6	2058.501	0.05
	Age^3^ * Sex + BQI	4.445	10	2049.311	0.12
	Age^3^ + BQI	4.507	6	2058.724	0.05
	Age^3^ + Sex + BQI	4.556	7	2056.501	0.07
	Age² * Sex	4.611	7	2056.556	0.07
	Age² * Sex + BQI	4.773	8	2054.403	0.08
	Age^3^ * Sex	5.115	9	2052.386	0.10
	Age² * BQI	6.093	7	2058.039	0.05
	Age² * BQI + Sex	6.442	8	2056.073	0.07
	Age^3^ * BQI	7.535	9	2054.806	0.08
	Age^3^ * BQI + Sex	7.813	10	2052.679	0.10
	*Undulations per g ~ Best intrinsic model* +/× *Extrinsic variables (n* = *114)*				
24	**Age + Year + StudyDay**	**0**	**7**	**2034.613**	**0.23**
25	**InvAge + Year + StudyDay**	**0.355**	**7**	**2034.968**	**0.23**
26	**Age + Year**	**1.186**	**6**	**2038.071**	**0.21**
27	**InvAge + Year**	**1.501**	**6**	**2038.386**	**0.20**
	Year + StudyDay*Age	2.199	8	2034.497	0.23
	Year + StudyDay + Sex	2.209	7	2036.822	0.21
	Year + StudyDay*InvAge	2.265	8	2034.563	0.23
	Year*Age + StudyDay	2.334	10	2029.869	0.26
	StudyDay + Year	2.787	6	2039.672	0.19
	Sex + Year	3.588	6	2040.473	0.19
	Year*Age	3.963	9	2033.902	0.23
	Year*InvAge + StudyDay	4.736	10	2032.27	0.24
	Year	4.941	5	2044.056	0.16
	Year*InvAge	6.186	9	2036.126	0.22
	Age	17.332	3	2060.784	0.03
	InvAge	17.589	3	2061.041	0.03
	Age + StudyDay	17.733	4	2059.036	0.04
	InvAge + StudyDay	17.993	4	2059.296	0.04
	StudyDay	18.422	3	2061.874	0.02
	Sex	18.448	3	2061.9	0.02
	Intercept only	18.658	2	2064.22	0.00
	Sex + StudyDay	19.027	4	2060.33	0.03
	StudyDay*Age	19.154	5	2058.269	0.05
	StudyDay*InvAge	19.513	5	2058.628	0.05

Only models performing better than the Intercept-only model and within 2 ΔAICc of the best model, in bold, are numbered. InvAge = −1/Age, BQI = Breeding Quality Index. *Lowest AICc* = *2067.002 for Model 15 and 2049.670 for Model 24*.

All statistics were performed using R 3.4.0^60^. Acceleration data were analysed with IGOR Pro 6 (WaveMetrics Inc., USA). Means ± SE are given unless indicated otherwise.

## Results

### Effects of Age on Foraging Efficiency

We found no evidence for a quadratic (i.e., #4 in Fig. 1) or cubic (#3 in Fig. 1) effect of age on either of the foraging efficiency proxies (Tables 1 and 2). Instead, all the top models included either a linear (#1 in Fig. 1) or a pseudo-threshold (#2 in Fig. 1) effect of age on foraging efficiency. For both Uperday (Table 2) and Uperg (Table 3), models including a linear (models 2 and 15) and a pseudo-threshold (models 1 and 16) effect of age were equally competitive, therefore supporting the hypothesis that foraging efficiency increases in younger age classes (maturation or progressive selection of more efficient individuals), then either continues to increase in older age classes or reaches a plateau.

For Uperday, the best model based on intrinsic characteristics only (Table 2, model 1) included a pseudo-threshold effect of age ($\hat{\beta }$ = 1453.8, SE = 580.3, 85% CI: 614.6 to 2293.0). According to this model, 5 yr-old birds performed 65 more undulations per day on average than 4 yr-old birds. This difference between successive age classes decreases with older age to reach only 5 undulations between 16 and 17 yr-old individuals. A linear effect of age (Table 2, model 2) was also supported ($\hat{\beta }$ = 23.4, SE = 9.6, 85% CI: 9.6 to 37.3), predicting an increase of 23 undulations per day with each year of age.

For Uperg, the best model based on intrinsic characteristics only (Table 3, model 15) included a linear effect of age ($\hat{\beta }$ = 108.0, SE = 58.4, 85% CI: 23.4 to 192.6). This model predicted an increase of 108 undulations per g for each year of increasing age. A pseudo-threshold effect of age (Table 3, model 16) was also supported ($\hat{\beta }$ = 6387.2, SE = 3589.3, 85% CI: 1184.7 to 11589.7).

### Effects of sex and Individual Quality on Foraging Efficiency

**Figure 3:**
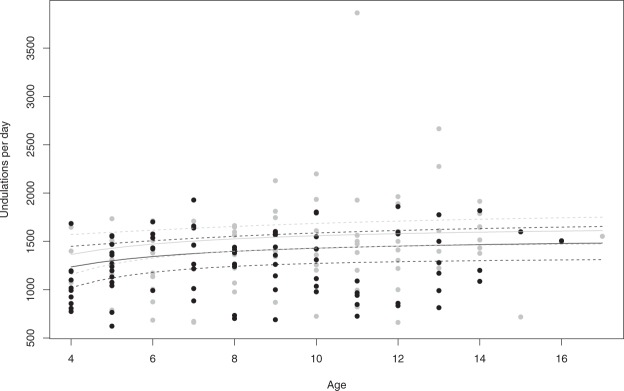
The number of undulations per day increases with age, faster in younger age classes, before reaching a plateau and is higher in males (in grey) than in females (in black). Linear regression equation and 95% CI from model 11: y = 1558.5 + 1292.7 * (−1/Age) + 128.9 * Sex(male) – 12.6 * Study Day(Dec 20); R² = 0.10. A linear increase of the number of undulations per day with age (model 12) is also supported by our data.

Higher foraging efficiency of male Adélie penguins relative to females was supported in terms of Uperday (Fig. 3) and male Adélie penguins performed 157 more undulations per day (Table 2, model 1, $\hat{\beta }$ = 157.2, SE = 61.5, 85% CI: 68.2 to 246.2) than their female counterparts. Sex was also included in 6 of the 9 top models based on intrinsic variables for Uperg (Table 3) but examination of the associated 85% CI suggested this was a weaker effect (model 17, $\hat{\beta }$ = 494.6, SE = 388.4, 85% CI: −68.3–1057.6; model 22, $\hat{\beta }$ = 587.5, SE = 387.2, 85% CI: 26.3–1148.8). Remaining competitive models containing the interaction between a linear or a pseudo-threshold effect of age and sex all contained uninformative covariates.

Higher foraging efficiency of higher quality individuals was not strongly supported. BQI was included in 2 of the 6 top models based on intrinsic variables for Uperday (Table 2) and in 2 of the 9 top models for Uperg (Table 3). However, in all models, the associated 85% CI included zero (e.g. model 4, $\hat{\beta }$ = 129.2, SE = 173.9, 85% CI: −122.3 to 380.7; model 19, $\hat{\beta }$ = 1435.4, SE = 1084.1, 85% CI: −136.2 to 3006.9). Interactions between BQI and age were not supported.

### Effects of Extrinsic Variables on Foraging Efficiency

**Figure 4:**
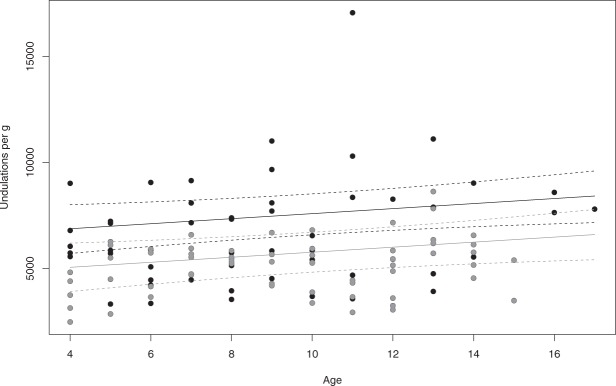
The number of undulations per g linearly increases with age and is higher in 2013 and 2010 (black dots) than in 2011 and 2012 (grey dots). Regression lines and 95% CI are drawn for 2013 (in black) and 2011 (in grey) from model 28 using the following equations: 6625.6 + 118.8 * Age – 225.5 * Year(2013); 6625.6 + 118.8 * Age – 2044.8 * Year(2011); R² = 0.23. A pseudo-threshold relationship between the number of undulations per day and age (model 23) is also supported by our data.

Uperday and Uperg significantly varied between years as shown by the presence of year in 4 of the 8 top models including extrinsic variables for Uperday (Table 2, models 9–12) and in all 4 top models for Uperg (Table 3, models 24–27). Uperday was slightly higher in 2012 than in all other years (model 9, $\hat{\beta }$ = 188.5, SE = 87.6, 85% CI: 61.9 to 315.2, with 2010 as the reference year when penguins performed 1121 undulations per day on average). Uperg was higher in 2010 (model 24, $\hat{\beta }$ = 6625.6, SE = 970.2, 85% CI: 5218.9 to 8032.2; Fig. 4) and 2013 ($\hat{\beta }$ = −225.5 compared to 2010, SE = 929.2, 85% CI: −1572.7 to 1121.7) than in 2011 ($\hat{\beta }$ = −2044.8 compared to 2010, SE = 920.7, 85% CI: −3379.7 to −709.9) and 2012 ($\hat{\beta }$ = −1914.9 compared to 2010, SE = 943.6, 85% CI: −3283.0 to −546.9). Interactions between year and age were not supported.

A negative effect of study day on foraging efficiency was included in 4 of the 8 top models including extrinsic variables for Uperday (Table 2, models 7–10) and 2 of the 4 top models for Uperg (Table 3, models 24–25). Accordingly, both Uperday (model 7, $\hat{\beta }$ = −12.6, SE = 7.1, 85% CI: −22.9 to −2.3) and Uperg (model 24, $\hat{\beta }$ = −79.7, SE = 43.7, 85% CI: −143.0 to −16.3) decreased as the season progressed. Interactions between study day and age were not supported.

## Discussion

We found support for a positive effect of age, either linear or levelling off at old ages, on both indices of foraging efficiency in breeding Adélie penguins. Younger birds exhibited lower foraging efficiency, both in terms of prey capture per unit of time (Uperday) and per unit of dynamic acceleration (Uperg). Other variables had different effects on these two indices. Across all age classes, males showed higher foraging efficiency than females in terms of Uperday but this effect was weaker for Uperg. Uperg was marked by strong inter-annual variations, while Uperday was more driven by an increase in trip duration throughout the breeding season (study day effect). Overall, extrinsic variables, year and study day, tended to explain more of the variation in foraging efficiency than age.

### Individual Improvement and Selective Disappearance of Phenotypes

Our results are consistent with both hypotheses (Hyp. 1 and 2) involving a continuous improvement of foraging efficiency and/or the progressive selection of more efficient individuals, possibly levelling off in older age classes. These two mechanisms will be difficult to tease apart and to quantify without a long-term, longitudinal study of foraging efficiency. However, it is very likely that both individual improvement and selective disappearance of individuals from the population play a role in the observed pattern. Adélie penguins live in an extremely harsh and selective environment and experience high predation rates^31^. In a previous study^35^, we showed the existence of demographic heterogeneity (i.e. some types of individuals having greater or lesser chances of surviving or reproducing)^61^ in the Cape Crozier population. We also showed that the first reproductive event was likely acting as a selective process leading to a more homogeneous pool of experienced breeders, highlighting the fact that some selective disappearance is indeed happening, at least during the early stages of reproductive life. On the other hand, recent studies on reproductive performance accounting for unobserved heterogeneity (see^62^ and references therein) showed that average individual improvement may be a widespread phenomenon in birds and mammals. In their study on common terns, Rebke *et al*.^62^ suggested that the observed age-specific patterns in average improvement in reproductive performance among survivors between age 3 and 14 yrs may be due to a gain in experience, possibly through improved foraging ability. Clearly, in brown pelicans, older birds have fewer accidents, i.e. taking water depth into consideration before plunging into the ocean, than do naïve ones^27^. Our results on Adélie penguins confirm the possibility of continuous improvement of foraging ability through old age classes.

We did not find strong support for a direct relationship between breeding quality and foraging efficiency. This is in accordance with our previous results, showing that this relationship is only apparent under harsh environmental conditions^42^, with this current study not including years of conditions as harsh as those encountered in 2001–2005, when giant icebergs were present in the foraging area. Our focus on breeding individuals, i.e. only a portion of the diversity of phenotypes at fledging, also likely restrained the range of inter-individual variation. Additionally, we found no evidence for different age-related variations according to breeding quality, i.e. low and high-quality breeders do not age differently, at least as far as foraging efficiency is concerned. Therefore, it seems that under “normal” environmental conditions, individuals of higher intrinsic quality do not exhibit significantly higher foraging efficiency than lower quality individuals or maintain better their foraging efficiency into old ages (no effect of the interaction between age and BQI). Altogether, these results suggest that within-individual improvement, more so than selective disappearance of underperforming phenotypes and at least in older ages, may be responsible for a large amount of the observed increase in foraging efficiency with age. Interestingly, a recent longitudinal demographic analysis using long-term data gathered at all 3 colonies of Ross Island from 1996 to 2013 (Kappes *et al*. In prep) does show that within-individual improvement is the main mechanism underlying patterns of age-related changes in reproductive performance at Cape Crozier. Given that the average age at first reproduction (shown to act as a selective process) is just under 6 yr old at Cape Crozier (Kappes *et al*. In prep), and that most of the observed variation in Uperday (but not in Uperg) occurs up to this age, it is possible that selective disappearance could be at least partially responsible of the observed improvement of foraging efficiency in early ages.

### Behavioral senescence

As in the most recent studies on age-related variation in foraging behaviour of seabirds^22,23^, we did not find any sign of senescence on global indices of foraging efficiency. On thick-billed murres, Elliott *et al*.^22^ were able to find signs of physiological senescence in parameters linked to diving capacity (haematocrit, resting metabolic rate and thyroid hormone levels), while Cunningham *et al*.^63^ detected signs of deterioration of the diving performance at the scale of the dive. The fact that it was possible to detect early signs of senescence at a very fine scale but not on the resulting foraging efficiency means that either old birds are able to adjust their behaviour to compensate for decreased fine-scale performance and/or that they die shortly after the onset of senescence. In common terns, only 20% of all mature birds reached the age when reproductive senescence may become important^62^. In Adélie penguins, high mortality levels due to predation^37^ and harsh environmental conditions may explain why we could not detect signs of senescence in foraging efficiency.

### Sex Differences in Foraging Efficiency

Sexual differences in foraging behaviour and provisioning rates have been reported for many seabird species, including species with no or little sexual dimorphism such as Adélie penguins. Such differences have been explained by a sex-specific allocation of foraging effort between parents and offspring^64^, differences in time spent defending the nest^65–67^, or spatial segregation due to intra-specific competition or habitat selection^68^. Here and in previous studies^42^, e.g.^69^, we showed that female Adélie penguins from Cape Crozier stayed longer at sea for the same amount of food caught (using the number of dive undulations) or brought back to the colony (using an automated weighing system). This pattern was also found at Béchervaise Island, where female Adélie penguins made longer foraging trips than males, ranged greater distances more frequently and consumed larger quantities of krill^70^. In Adélie Land, however, Angelier *et al*.^71^ did not find any significant sex difference in foraging success, trip duration, or maximal foraging range, although females tended to range farther than males (94.72 ± 6.44 *vs*. 65.20 ± 16.46 km).

In our study colony, males bring back the same amount of food as females after shorter foraging trips but do not make more frequent trips or feed their chicks more frequently^42^. It seems therefore unlikely that the observed sex differences would stem from a sex-specific allocation of foraging effort between parents and offspring. Rather, it appears that male and female foraging areas are spatially segregated, with females going further away from the colony on longer trips and diving shallower^42^, while males exploit waters closer to the colony by diving deeper, thus allowing them to spend more time defending their territory. Interestingly, we found a much weaker effect of sex on Uperg than Uperday, suggesting that if the male strategy was indeed more efficient in terms of number of prey captures per unit of time spent at sea (Uperday), it was about as costly in terms of mechanical work (deep dives for males *vs*. longer trips for females). In an optimal foraging theory framework^72^, it seems that male Adélie penguins tend to maximize net energy gain per unit time, while females tend to maximize net energy gain per energy spent. However, more work is needed to examine fine-scale foraging habitat and diet preferences of males and females so as to determine whether this difference in foraging habitat use is based on based on niche specialization^68^, competitive exclusion^73^, or differences in nutritional requirements^74^.

We also acknowledge that our index of foraging success, i.e. the number of undulations in the dive profile, while linearly related to the number of prey captures in penguins, is an imperfect measure and could represent vastly different energy intake depending on whether it relates to crystal krill capture (*Euphausia crystallorophias*, average individual wet weights for subadults to adults:0.02 to 0.2 g, average individual energy content: 0.05 to 0.46 kJ)^75–77^ or silverfish (*Pleuragramma antarcticum*, average individual wet weight for older subadults: 3.42 g, average individual energy content: 8.92 kJ)^75,78^, the two main prey of Adélie penguins in the southern Ross Sea^79^. An individual foraging primarily on silverfish might undulate significantly less (in another study, Watanabe & Takahashi^46^ showed that Adélie penguins catch about 7 krill individuals for 1 fish *Pagothenia borchgrevinki*) and still gain the same amount of energy. However, krill is by far the dominant item of the diet of breeding Adélie penguins during the early chick-rearing season^57,75^, when we equipped them in this study. Additionally, the average number of undulations per trip is 2403 ± 1151 (this study) and average food load (i.e. food brought back to the chicks by a parent) is 592 ± 285 g^50^. This translates into 0.25 g per undulation. If we account for 15% of false positives (i.e. undulations without actual prey capture)^78^, it gives 2043 successful undulations and 0.29 g per undulation. Both estimates are consistent with a krill-dominated diet.

### Drivers of Foraging Efficiency

In our study, intrinsic characteristics of the birds, such as age, sex, or breeding quality explained a maximum of 6% (for Uperg) to 10% (for Uperday) of the variability in foraging efficiency (3 to 5% for the age only), compared to 9% (for Uperday) to 18% (for Uperg) for extrinsic variables (year, study day) only (Tables 2 and 3). We confirmed that foraging efficiency of birds of both sexes decreased as the season progressed, likely related to resources becoming depleted or inaccessible close to the very large Cape Crozier colony^54,55,57^. We identified large inter-annual variations in average foraging efficiency as proxied by Uperg, which may at least partially be driven by inter-annual variations in sea ice concentration (SIC) within the summer foraging area of Adélie penguins. At our study colony, maximum foraging efficiency during summer is known to occur at relatively low SIC, peaking at 6–12% and decreasing with higher SIC^80^. In 2011, the average SIC over monitored penguin foraging days was especially high (18.4%) and coincided with low foraging efficiency as well as an attenuation of the age effect. Under high SIC (13–38%), foraging efficiency is constrained to low levels^80^ as penguins have to walk over extended, continuous stretches of ice, or hop from floe to floe to avoid being crushed or attacked by predators hunting in the leads. In these conditions, foraging efficiency becomes essentially uncoupled from SIC variations as phenotypic plasticity is suppressed^80^, older age does not seem to provide a significant advantage anymore, but inter-individual differences linked to breeding quality are exacerbated^50^. Interestingly, the fact that Uperg was marked by stronger inter-annual variations than Uperday suggests that Adélie penguins first respond to more difficult environmental conditions by intensifying their foraging effort before increasing their trip duration, which can only be stretched to a given extent before chick survival is jeopardized.

As the amount of variation explained has rarely been reported in previous studies about the effect of age on foraging efficiency, it is difficult to put our results in perspective. As an indication, Elliott *et al*.^22^ report R² of 0.19 to 0.29 for the effect of age on physiological parameters in thick-billed murres, and Cunningham *et al*.^63^ report R² of 0.14 to 0.46 for the effect of age on energy expenditure (reported as Partial Dynamic Body Acceleration) during different parts of the dive in the same species. Compared to these, the 3–5% variation attributed to age in this study appears to be small but is in accord with behavioural traits generally being more labile than physiological or morphological ones^81^. Seabird behaviour in particular is sensitive to the variations in environmental conditions (hence their use as ecological indicators^82^ and also very plastic so that shifts in phenotypic plasticity can affect or blur the functional relationships between behavioural traits and environmental variables^80,83^. It is therefore not too surprising that age would account for only a small part of the variation in foraging efficiency, especially measured over 4 different years. Assuming that one undulation represents the capture of 0.29 g of food (at the minimum, not taking into account prey digestion during the early part of the trip), then age alone explains differences in prey catch up to 86.71 g (or 14.7% of the total food load) per day between the youngest and oldest birds in our study. Under competitive conditions at large colonies such as Cape Crozier^47,54,57^, even a small advantage due to age or intrinsic “quality” may make a difference in terms of reproductive success and/or survival.

## Data Availability

Data collected are available at California Avian Data Center (CADC) hosted by Point Blue Conservation Science and metadata registered with the “Antarctic Master Directory” (http://gcmd.nasa.gov/KeywordSearch/Home.do?Portal=amd&MetadataType=0). Data are and will be available at CADC: http://data.prbo.org/apps/penguinscience/.

## References

[1] Clutton-Brock, T. H. *Reproductive success: studies of individual variation in contrasting breeding systems*. (University of Chicago Press, 1988).

[2] Newton, I. & others. *Lifetime reproduction in birds*. (Academic Press, 1989).

[3] Massot M (2011). An integrative study of ageing in a wild population of common lizards. Funct. Ecol..

[4] Curio E (1983). Why de young birds reproduce less well?. Ibis.

[5] Forslund P, Pärt T (1995). Age and reproduction in birds—hypotheses and tests. Trends Ecol. Evol..

[6] Hamilton WD (1966). The moulding of senescence by natural selection. J. Theor. Biol..

[7] Williams GC (1957). Pleiotropy, natural selection, and the evolution of senescence. Evolution.

[8] Kirkwood TB (1977). Evolution of ageing. Nature.

[9] Cam E, Link WA, Cooch EG, Monnat J-Y, Danchin E (2002). Individual covariation in life-history traits: seeing the trees despite the forest. Am. Nat..

[10] Kirkwood TB, Austad SN (2000). Why do we age?. Nature.

[11] Stearns, S. C. *The evolution of life histories*. **249** (Oxford University Press Oxford, 1992).

[12] Descamps S, Boutin S, Berteaux D, GAILLARD J-M (2007). Female red squirrels fit Williams’ hypothesis of increasing reproductive effort with increasing age. J. Anim. Ecol..

[13] Heidinger BJ, Nisbet IC, Ketterson ED (2006). Older parents are less responsive to a stressor in a long-lived seabird: a mechanism for increased reproductive performance with age?. Proc. R. Soc. Lond. B Biol. Sci..

[14] Angelier F, Weimerskirch H, Dano S, Chastel O (2007). Age, experience and reproductive performance in a long-lived bird: a hormonal perspective. Behav. Ecol. Sociobiol..

[15] Palacios MG, Cunnick JE, Winkler DW, Vleck CM (2007). Immunosenescence in some but not all immune components in a free-living vertebrate, the tree swallow. Proc. R. Soc. Lond. B Biol. Sci..

[16] Cheynel L (2017). Immunosenescence patterns differ between populations but not between sexes in a long-lived mammal. Sci. Rep..

[17] Catry P, Phillips RA, Phalan B, Croxall JP (2006). Senescence effects in an extremely long-lived bird: the grey-headed albatross Thalassarche chrysostoma. Proc. R. Soc. Lond. B Biol. Sci..

[18] Catry P, Granadeiro JP, Ramos J, Phillips RA, Oliveira P (2011). Either taking it easy or feeling too tired: old Cory’s Shearwaters display reduced activity levels while at sea. J. Ornithol..

[19] Daunt F, Wanless S, Harris MP, Money L, Monaghan P (2007). Older and wiser: improvements in breeding success are linked to better foraging performance in European shags. Funct. Ecol..

[20] Limmer B, Becker PH (2009). Improvement in chick provisioning with parental experience in a seabird. Anim. Behav..

[21] Lecomte VJ (2010). Patterns of aging in the long-lived wandering albatross. Proc. Natl. Acad. Sci..

[22] Elliott KH (2015). Ageing gracefully: physiology but not behaviour declines with age in a diving seabird. Funct. Ecol..

[23] Froy H (2015). Age-related variation in foraging behaviour in the wandering albatross at South Georgia: no evidence for senescence. PloS One.

[24] MacNulty DR (2009). Predatory senescence in ageing wolves. Ecol. Lett..

[25] Hassrick JL, Crocker DE, Costa DP (2013). Effects of maternal age and mass on foraging behaviour and foraging success in the northern elephant seal. Funct. Ecol..

[26] Froy, H. *et al*. Declining home range area predicts reduced late-life survival in two wild ungulate populations. *Ecol. Lett* (2018). 10.1111/ele.12965 29656580

[27] Orians GH (1969). Age and hunting success in the Brown Pelican (Pelecanus occidentalis). Anim. Behav..

[28] Jones OR (2008). Senescence rates are determined by ranking on the fast–slow life-history continuum. Ecol. Lett..

[29] Jouventin P, Lequette B, Dobson FS (1999). Age-related mate choice in the wandering albatross. Anim. Behav..

[30] Dugger KM, Ainley DG, Lyver PO, Barton K, Ballard G (2010). Survival differences and the effect of environmental instability on breeding dispersal in an Adélie penguin meta-population. Proc. Natl. Acad. Sci..

[31] Ainley, D. *The Adélie penguin: bellwether of climate change*. (Columbia University Press, 2002).

[32] Ballard G (2010). Responding to climate change: Adélie penguins confront astronomical and ocean boundaries. Ecology.

[33] Clarke J (2003). Post-fledging and winter migration of Adélie penguins Pygoscelis adeliae in the Mawson region of East Antarctica. Mar. Ecol. Prog. Ser..

[34] Hinke JT (2015). Spatial and isotopic niche partitioning during winter in chinstrap and Adélie penguins from the South Shetland Islands. Ecosphere.

[35] Lescroël A, Dugger KM, Ballard G, Ainley DG (2009). Effects of individual quality, reproductive success and environmental variability on survival of a long-lived seabird. J. Anim. Ecol..

[36] Lyver PO (2014). Trends in the breeding population of Adélie penguins in the Ross Sea, 1981–2012: a coincidence of climate and resource extraction effects. PLoS One.

[37] Ainley DG, Ballard G, Karl BJ, Dugger KM (2005). Leopard seal predation rates at penguin colonies of different size. Antarct. Sci..

[38] Ainley DG, Ballard G (2012). Non-consumptive factors affecting foraging patterns in Antarctic penguins: a review and synthesis. Polar Biol..

[39] Dugger KM, Ballard G, Ainley DG, Barton KJ (2006). Effects of flipper bands on foraging behavior and survival of Adélie penguins (Pygoscelis adeliae). The Auk.

[40] Wilson, R. P. & Wilson, M.-P. T. Tape: a package-attachment technique for penguins. *Wildl. Soc. Bull*. 77–79 (1989).

[41] Ballard G, Ainley DG, Ribic CA, Barton KR (2001). Effect of instrument attachment and other factors on foraging trip duration and nesting success of Adélie penguins. The Condor.

[42] Lescroël A (2010). Working less to gain more: when breeding quality relates to foraging efficiency. Ecology.

[43] Ropert-Coudert Y (2001). Feeding strategies of free-ranging Adélie penguins Pygoscelis adeliae analysed by multiple data recording. Polar Biol..

[44] Bost C-A (2007). Changes in dive profiles as an indicator of feeding success in king and Adélie penguins. Deep Sea Res. Part II Top. Stud. Oceanogr..

[45] Takahashi A (2004). Krill-feeding behaviour in a chinstrap penguin compared to fish-eating in Magellanic penguins: a pilot study. Mar. Ornithol..

[46] Watanabe YY, Takahashi A (2013). Linking animal-borne video to accelerometers reveals prey capture variability. Proc. Natl. Acad. Sci..

[47] Ballance LT, Ainley DG, Ballard G, Barton K (2009). An energetic correlate between colony size and foraging effort in seabirds, an example of the Adélie penguin Pygoscelis adeliae. J. Avian Biol..

[48] Wilson RP (2006). Moving towards acceleration for estimates of activity-specific metabolic rate in free-living animals: the case of the cormorant. J. Anim. Ecol..

[49] Halsey LG (2011). Assessing the Validity of the Accelerometry Technique for Estimating the Energy Expenditure of Diving Double-Crested Cormorants Phalacrocorax auritus. Physiol. Biochem. Zool..

[50] Shepard EL (2008). Derivation of body motion via appropriate smoothing of acceleration data. Aquat. Biol..

[51] Shepard EL, Wilson RP, Laich AG, Quintana F (2010). Buoyed up and slowed down: speed limits for diving birds in shallow water. Aquat. Biol..

[52] Fridolfsson, A.-K. & Ellegren, H. A simple and universal method for molecular sexing of non-ratite birds. *J. Avian Biol*. 116–121 (1999).

[53] Grémillet, D. *et al*. Energetic fitness: Field metabolic rates assessed via 3D accelerometry complement conventional fitness metrics. *Funct. Ecol* (In press).

[54] Ainley DG (2004). Geographic structure of Adélie Penguin populations: Overlap in colony-specific foraging areas. Ecol. Monogr..

[55] Ainley, D. G. *et al*. Trophic cascades in the western Ross Sea, Antarctica: revisited (2015). 10.1890/0012-9658(2006)87[2080:capacr]2.0.co;2 16937647

[56] Ford RG (2015). Testing assumptions of central place foraging theory: a study of Adélie penguins Pygoscelis adeliae in the Ross Sea. J. Avian Biol..

[57] Ainley DG, Ballard G, Dugger KM (2006). Competition among penguins and cetaceans reveals trophic cascades in the western Ross Sea, Antarctica. Ecology.

[58] Burnham, K. P. & Anderson, D. R. *Model selection and multimodel inference: a practical information-theoretic approach*. (2002).

[59] Arnold TW (2010). Uninformative parameters and model selection using Akaike’s InformationCriterion. J. Wildl. Manag..

[60] R Development Core Team. *R: A Language and Environment for Statistical Computing*. (R Foundation for Statistical Computing, 2017).

[61] Fox GA, Kendall BE, Fitzpatrick JW, Woolfenden GE (2006). Consequences of heterogeneity in survival probability in a population of Florida scrub-jays. J. Anim. Ecol..

[62] Rebke M, Coulson T, Becker PH, Vaupel JW (2010). Reproductive improvement and senescence in a long-lived bird. Proc. Natl. Acad. Sci..

[63] Cunningham JT (2017). Reduced activity in middle-aged thick-billed murres: evidence for age related trends in fine-scale foraging behaviour. Anim. Behav..

[64] Thaxter CB (2009). Sex-specific food provisioning in a monomorphic seabird, the common guillemot Uria aalge: nest defence, foraging efficiency or parental effort?. J. Avian Biol..

[65] Wanless S, Harris MP (1986). Time spent at the colony by male and female guillemots Uria aalge and razorbills Alca torda. Bird Study.

[66] Creelman, E. & Storey, A. E. Sex differences in reproductive behavior of Atlantic Puffins. *Condor* 390–398 (1991).

[67] Fraser GS, Jones IL, Hunter FM (2002). Male-female differences in parental care in monogamous crested auklets. The Condor.

[68] Cleasby IR (2015). Sexual segregation in a wide-ranging marine predator is a consequence of habitat selection. Mar. Ecol. Prog. Ser..

[69] Ballard G, Dugger KM, Nur N, Ainley DG (2010). Foraging strategies of Adélie penguins: adjusting body condition to cope with environmental variability. Mar. Ecol. Prog. Ser..

[70] Clarke J (1998). Sex differences in Adélie penguin foraging strategies. Polar Biol..

[71] Angelier F (2008). Corticosterone and foraging behavior in a diving seabird: the Adélie penguin, Pygoscelis adeliae. Gen. Comp. Endocrinol..

[72] Stephens, D. W. & Krebs, J. R. *Foraging theory* (Princeton University Press, 1986).

[73] Phillips RA, Silk JRD, Phalan B, Catry P, Croxall JP (2004). Seasonal sexual segregation in two Thalassarche albatross species: competitive exclusion, reproductive role specialization or foraging niche divergence?. Proc. R. Soc. B Biol. Sci..

[74] Lewis S (2002). Sex-specific foraging behaviour in a monomorphic seabird. Proc. R. Soc. Lond. B Biol. Sci..

[75] Ainley DG (2003). Spatial and temporal variation of diet within a presumed metapopulation of Adélie penguins. The Condor.

[76] Ju S-J, Harvey HR (2004). Lipids as markers of nutritional condition and diet in the Antarctic krill Euphausia superba and Euphausia crystallorophias during austral winter. Deep Sea Res. Part II Top. Stud. Oceanogr..

[77] Wiebe PH, Boyd S, Cox JL (1975). Functional regression equations for zooplankton displacement volume, wet weight, dry weight, and carbon. Fish. Bull..

[78] Mayzaud P, Chevallier J, Tavernier E, Moteki M, Koubbi P (2011). Lipid composition of the Antarctic fish Pleuragramma antarcticum. Influence of age class. Polar Sci..

[79] Ainley DG (1998). Diet and foraging effort of Adélie penguins in relation to pack-ice conditions in the southern Ross Sea. Polar Biol..

[80] Lescroël A, Ballard G, Grémillet D, Authier M, Ainley DG (2014). Antarctic climate change: extreme events disrupt plastic phenotypic response in Adélie penguins. PloS One.

[81] Blomberg SP, Garland T, Ives AR (2003). Testing for phylogenetic signal in comparative data: behavioral traits are more labile. Evolution.

[82] Piatt JF (2007). Seabirds as indicators of marine food supplies: Cairns revisited. Mar. Ecol. Prog. Ser..

[83] Grémillet D, Charmantier A (2010). Shifts in phenotypic plasticity constrain the value of seabirds as ecological indicators of marine ecosystems. Ecol. Appl..

